# O9-7 Community-based physical activity promotion for mental health and wellbeing in ‘hard-to-reach' groups

**DOI:** 10.1093/eurpub/ckac094.071

**Published:** 2022-08-29

**Authors:** Niamh Murphy, Annemarie Wagemakers, Kirsten Verkooijen, Aisling McGrath, Evan Matthews

**Affiliations:** 1 Dept of Sport and Exercise Science, Waterford Institute of Technology, Waterford, Ireland; 2 Department of Social Sciences, Wageningen University, Wageningen, The Netherlands

**Keywords:** physical activity, mental health, community interventions, social disadvantage

## Abstract

Community-based health enhancing physical activity (PA) initiatives often lead to important mental health and wellbeing outcomes beyond improvements in physical activity levels. However, identifying what outcomes to measure and how to measure them is a challenge in community-based work. There is a lack of practical guidance on how to strengthen mental health and wellbeing outcomes within the context of PA initiatives. This presentation will share the work on a project with community based practitioners and mental health/physical activity experts which is being led by the HEPA Europe Working Group on HEPA promotion in socially disadvantaged groups, supported by WHO Europe.

The presentation will engage participants in discussions around the following questions:

1. What are the most important mental health and wellbeing outcomes of community-based health enhancing physical activity initiatives in Europe, and how can these best (i.e., adequately, and pragmatically) be assessed for evaluation purposes?
2. What are the needs of practitioners (from physical activity sectors and mental health sectors) working in communities to promote mental health through physical activity?

